# Association between central thyroid hormone sensitivity and prediabetes: Tehran thyroid study

**DOI:** 10.3389/fendo.2025.1534058

**Published:** 2025-06-17

**Authors:** Ladan Mehran, Mohammadjavad Honarvar, Maryam Tohidi, Maryam Adib, Fereidoun Azizi, Atieh Amouzegar

**Affiliations:** ^1^ Endocrine Research Center, Research Institute for Endocrine Disorders, Research Institute for Endocrine Sciences, Shahid Beheshti University of Medical Sciences, Tehran, Iran; ^2^ Prevention of Metabolic Disorders Research Center, Research Institute for Endocrine Sciences, Shahid Beheshti University of Medical Sciences, Tehran, Iran

**Keywords:** thyroid function, parametric thyroid feedback quantile-based index, thyroid hormone resistance, prediabetes, thyroid-stimulating hormone index, thyrotrophic thyroxin resistance index

## Abstract

**Background:**

Thyroid hormone sensitivity indices represent a recently proposed clinical entity related to metabolic health outcomes. The link between thyroid hormone sensitivity and prediabetes is not clear. This population-based study investigated the association between thyroid hormone sensitivity indices and prediabetes.

**Methods:**

Among 5,783 participants over 20 years, after excluding those receiving thyroid medications or corticosteroid drugs, having thyroid surgery, having a history of cancer, pregnant women, and those with end-stage renal disease, 4,356 subjects were included in the study. The odds ratio (OR) and 95% confidence interval (95%CI) for prediabetes in the general and euthyroid population per 1-SD increase in thyroid hormone resistance indices (PFTQI, TSHI, and lnTT4RI) were reported with logistic regression models.

**Results:**

One SD increase in PTFQI was significantly associated with lower odds of prediabetes even after total adjustment (OR:0.88; 95%CI: 0.82–0.94). The association was observed in women, non-smokers, and those with negative anti-thyroid peroxidase antibodies. In the euthyroid subgroup, one SD increase in PTFQI, TSHI, and lnTT4RI showed lower odds of prediabetes and [PTFQI: 0.89 (95%CI: 0.83–0.97); TSHI: 0.83 (95%CI: 0.74–0.94); lnTT4RI: 0.83 (95%CI: 0.74–0.93)]. We also found a negative correlation between thyroid hormone sensitivity indices and fasting plasma glucose (PTFQI: r =-0.094, TSHI:-0.1, and lnTT4RI: r =-0.098) and 2-h post-challenge glucose (PTFQI: r =-0.096, TSHI:-0.054, and lnTT4RI: r =-0.031).

**Conclusion:**

Lower central sensitivity to thyroid hormone, as represented by increased TSHI, TT4RI, and PTFQI, is associated with a lower risk of prediabetes, even in euthyroid individuals.

## Graphical Abstract

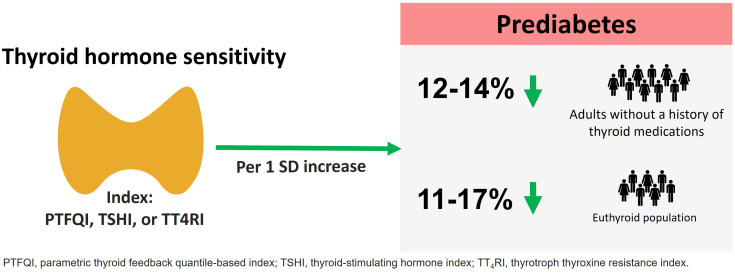

## Introduction

Prediabetes is a high-risk metabolic state characterized by intermediate hyperglycemia, affecting over 400 million adults worldwide (1, 2). Without intervention, nearly half of affected individuals progress to type 2 diabetes within five years, while also facing elevated risks of cardiovascular, renal, and neurological complications (3–6).

Thyroid hormones regulate glucose metabolism by modulating insulin sensitivity (via GLUT4 in muscle/fat) and hepatic glucose output (via PEPCK/G6Pase). Both hypothyroidism and hyperthyroidism have been suggested to influence insulin and glucose metabolism (7). Limited studies have explored the association between thyroid function and dysglycemia, and the results have been somewhat contradictory. A meta-analysis of 9 studies found a J-shaped relationship between thyroid-stimulating hormone (TSH, also known as thyrotropin) and free thyroxin (FT4) levels and diabetes (8). Thus, a more complex pathophysiological mechanism may drive the relationship between thyroid function and glucose homeostasis.

Elevated thyroid hormones and TSH suggest an acquired resistance to thyroid hormones in the general population, which may be present in those with normal thyroid function. Thyroid hormone sensitivity indices including parametric thyroid feedback quantile-based index (PTFQI) (9), thyrotrophic thyroxin resistance index (TT4RI) (10), and thyroid-stimulating hormone index (TSHI) (11) are valuable tools for assessing the sensitivity of peripheral tissues and the pituitary gland to thyroid hormones. They are particularly useful in conditions where standard thyroid function tests (TSH, fT4, fT3) may not fully reflect tissue-level thyroid hormone action, such as in resistance to thyroid hormone (RTH), central hypothyroidism, or non-thyroidal illness (NTI).

Despite the growing global burden of prediabetes, the role of thyroid hormone sensitivity - a potentially modifiable yet frequently overlooked metabolic factor - remains poorly understood in current clinical practice. While traditional risk factors like obesity and physical inactivity dominate prediabetes research, endocrine dysregulation (particularly thyroid function) offers a promising but underexplored avenue for early intervention. To date, most studies have focused on overt thyroid disease or isolated TSH/fT4 levels, with little attention to hypothalamic-pituitary-thyroid axis resistance—particularly in euthyroid individuals.

The investigation of thyroid hormone sensitivity in prediabetes is motivated by three critical evidence gaps in existing literature. First, while numerous studies have established associations between overt thyroid dysfunction (hypo-/hyperthyroidism) and diabetes risk (6, 12), the role of *subclinical* variations in thyroid hormone sensitivity—particularly within euthyroid ranges—remains poorly understood. Second, emerging research suggests that traditional thyroid markers (TSH/FT4) alone cannot fully explain metabolic dysregulation, as demonstrated by the J-shaped relationship between thyroid function and diabetes incidence (12). This paradox highlights the need to evaluate more sophisticated indices like PTFQI and TSHI that quantify central resistance to thyroid hormones (11, 13). Third, recent population studies reveal that even mild thyroid hormone resistance (reflected by elevated TSHI or TT4RI) is associated with altered glucose metabolism (9), yet no consensus exists on whether this relationship holds for prediabetes—a critical window for diabetes prevention. Our study directly addresses these gaps by examining thyroid sensitivity indices in a large, well-characterized cohort, thereby clarifying whether thyroid hormone resistance represents an independent risk factor or compensatory mechanism in early dysglycemia while stratifying for key subgroups (sex, smoking, autoimmunity).

## Methods

### Study design and study population

**Figure 1:**
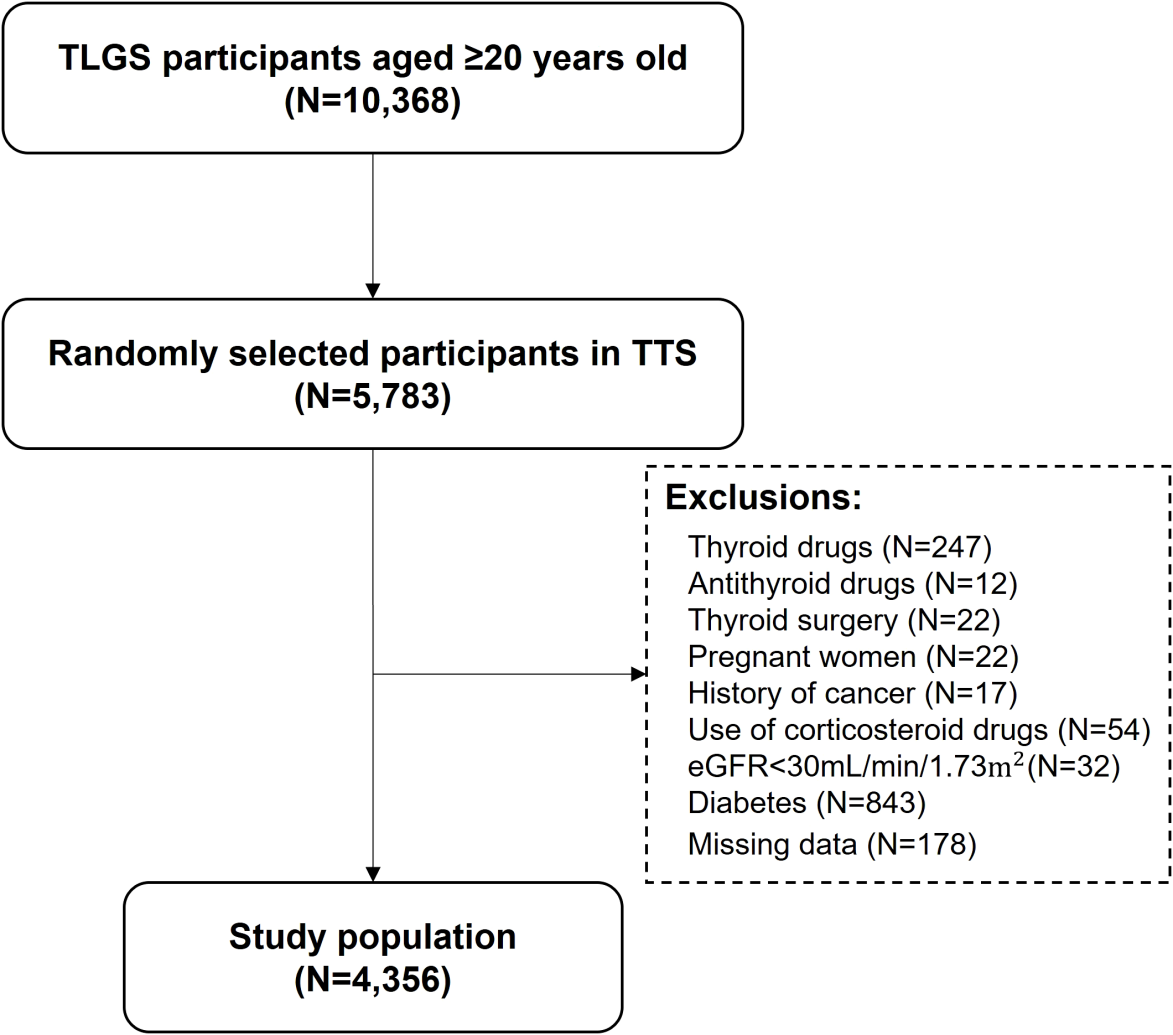
Flow chart of the study participants. Participants from the Tehran Lipid and Glucose Study (TLGS) were randomly selected for participation in the Tehran Thyroid study to investigate Thyroid diseases and their risk factors. After excluding individuals based on predefined criteria such as missing data or pre-existing conditions, the final study population was included for analysis. Abbrev: TLGS, Tehran Lipid and Glucose Study; TTS, Tehran Thyroid Study; eGFR, estimated glomerular filtration rate.

Tehran Thyroid Study (TTS) is a prospective population-based cohort study of the residents of District 13 of Tehran over 10 years, in 4 consecutive follow-ups from 1999–2009 within the Tehran Lipid and Glucose Study (TLGS) framework (14). The design and methodology of the TTS have been previously reported. A total of 5769 adults aged ≥ 20 years were selected using a random sampling method The current study is a cross-sectional analysis of baseline data (1999–2001) from the TTS cohort (15). The exclusion criteria in this study were the use of thyroid medications (n = 247), the use of antithyroid drugs (n = 12), thyroid surgery (n = 22), pregnant women (n = 22), history of cancer (n = 17), history of diabetes (n=843), use of corticosteroid drugs (n = 54), estimated glomerular filtration rate <30 mL/min/1.73m^2^ (n = 32), and missing data on covariates (n = 178). Finally, 4,356 subjects were included in this study (**Figure 1**).

### History and clinical examination

Trained healthcare professionals used the predesigned questionnaire to collect basic demographics, drug history, family and personal history of thyroid diseases, radioactive iodine intake, education level, physical activity levels, and smoking habits. Physical activity was assessed by the Lipid Research Clinic (LRC) and the Modifiable Activity Questionnaires (MAQ) and reported in the metabolic equivalent of the task (MET) scale; participants with less than 600 MET per week of activity were categorized as individuals with low physical activity. Education level categories were defined as follows considering the degree and years of education: primary school (less than six years), high school (6–12 years), and higher education (>12 years). Current smokers were defined as the occasional or daily use of tobacco. Baseline physical examinations, including blood pressure (BP) and anthropometric measurements, were performed by trained physicians. BP was recorded as a mean of two BP measurements on the right arm and at the heart level after participants remained seated for 15 minutes. After removing shoes and being minimally clothed, anthropometric measurements were recorded. Digital scales and tape meters were used to measure weight and height. Waist circumference (WC) was measured at the narrowest waist level. Body mass index (BMI) was calculated by dividing weight (kg) by the square of the height (m).

### Laboratory measurements

For all participants, after 12–14 hours of fasting overnight, blood samples were drawn between 7:00-9:00 AM. Within 30–45 minutes after collecting samples, they were centrifuged. Total cholesterol (TC), high-density lipoprotein cholesterol (HDL-C), fasting plasma glucose (FPG), and triglycerides (TG) were assayed. We performed the 75-gram oral glucose tolerance test (OGTT) for participants not taking glucose-lowering medications. The enzymatic colorimetric glucose oxidase method was used to assess fasting and 2-hour glucose concentrations. Serum TC and TG levels were recorded using the enzymatic calorimetric method with cholesterol esterase, cholesterol oxidase, and glycerol phosphate oxidase, respectively. Precipitation of the apolipoprotein B with lipoproteins and phosphotungstic acid was used to measure HDL-C.

An electrochemiluminescence immunoassay (ECLIA) with a Roche Diagnostics kit and a Roche/Hitachi Cobas e-411 analyzer (Roche GmbH, Mannheim, Germany) was used to measure TSH and FT4. An immunoenzymometric assay (IEMA) using a Sunrise ELISA reader (Tecan Co., Salzburg, Austria) was used to determine thyroid peroxidase antibodies (TPO-Ab).

### Definitions

Prediabetes was diagnosed based on the American Diabetes Association (ADA) 2023 guidelines, which define prediabetes as: Fasting plasma glucose (FPG) levels of 100–125 mg/dL (5.6–6.9 mmol/L) [impaired fasting glucose (IFG)], or 2-hour post-challenge glucose (2hPCG) levels of 140–199 mg/dL (7.8–11.0 mmol/L) after a 75-g oral glucose tolerance test (OGTT) [impaired glucose tolerance (IGT) (16). Euthyroidism was defined as the absence of thyroid dysfunction, including both clinical and subclinical forms of hypothyroidism and hyperthyroidism, using the reference ranges of TSH (0.32-5.06 mU/L) and fT4 (0.91-1.55 ng/dL) derived from the current population.

TT4RI was calculated as fT4 (pmol/L) multiplied by TSH (mU/L) (10), while the TSHI was determined as ln TSH (mU/L) + 0.1345 × fT4 (pmol/L) (11). Additionally, PTFQI, a measure of thyroid hormone resistance, evaluates the pituitary’s sensitivity to thyroid hormones by assessing the relationship between TSH and free thyroxine (fT4), often derived from population-based quantile regression models. It was calculated as cdf fT4 − (1 − cdf TSH), where the cumulative distribution function (cdf) represents a probability function (17). This calculation was based on specific reference values for fT4 and TSH for the Iranian population and can be easily computed using basic spreadsheet formulas.

We also defined categories of non-obese (BMI<30kg/m^2^) and obese (BMI≥30kg/m^2^), normal and low HDL-C (< 40 mg/dl for men and < 50 mg/dL for women), normal and high TG (>150 mg/dL), normal and high TC (>200 mg/dL) and negative and positive anti-TPO (TPOAb> 35 IU/mL for females and TPOAb> 32 IU/mL for males) (18) for categorical adjustments in multivariate logistic regression analysis.

### Statistical analysis

The baseline characteristics of participants were reported using mean ± standard deviation (SD) for continuous variables with normal distribution, median (interquartile range, IQR) for continuous variables with skewed distribution, and frequencies (%) for categorical variables. The normality of data was assessed using Kolmogorov-Smirnov (K-S) test and histograms with normally fitted curves. One-way analysis of variance (ANOVA), Kruskal-Wallis, and Chi-square tests were used to compare baseline characteristics of participants between the tertiles of PFTQI, TSHI, and TT4RI indices using means, medians, and frequencies, respectively.

Due to right-skewed distributions, TT4RI was natural log-transformed (lnTT4RI) for regression analyses. Results reflect ORs per 1-SD increase in lnTT4RI; back-transformed values are interpretable as multiplicative effects on the original scale.” We used multivariate logistic regression models to evaluate the association between thyroid hormone resistance indices (PFTQI, TSHI, and lnTT4RI) and prediabetes with both continuous and quartile approaches. The odds ratio (OR) and 95% confidence interval (95% CI) for prediabetes were reported for a 1-SD increase in thyroid sensitivity indices using the continuous approach and in comparison with the first tertile as the reference category in the tertile-based approach. Four models were defined: model 1 was adjusted for age and sex, model 2 was further adjusted for anti-TPO, model 3 was further adjusted for education level, smoking status, family history of diabetes, BMI, and physical activity, and model 4 was further adjusted for HDL-C, TG, and TC. In the tertile-based approach, the covariates in the models were adjusted categorically: anti-TPO (negative, positive), obesity (non-obese, obese), HDL-C (low, normal), TG (normal, high), and TC (normal, high). Moreover, the ORs for risk of prediabetes were examined in the euthyroid population. Finally, subgroup analysis of a 1-SD increase in thyroid hormone resistance indices based on sex (male or female), age (<45 or ≥45 years), anti-TPO (negative or positive), smoking (no or yes), and obesity (non-obese or obese) was performed. The correlation between thyroid hormone sensitivity indices and FPG and 2-hPCPG was also examined using the Spearman correlation. The statistical analysis was performed using STATA 17 (StataCorp, College Station, TX, USA) and R-3.0.3 (R Foundation for Statistical Computing, Vienna, Austria). Two-sided p-values less than 0.05 were considered statistically significant.

## Results

### Baseline characteristics of the study population

The baseline characteristics of the 4,356 study participants (43.0% male), with a mean age of 46.4 ± 13.7 years, are presented according to the PTFQI (**Table 1**), TSHI (**Supplementary Table 1**), and TT4RI tertiles (**Supplementary Table 2**). Participants in the highest tertile of PTFQI were younger, had lower WC, and had lower SBP, TG, and TC levels (p-value<0.001). TSH level was 2.1 (IQR: 1.4-3.4) mIU/L in the total population and 1.4 (IQR: 1.0-1.8), 2.7 (IQR: 1.7-4.4), and 2.9 (IQR: 2.1-4.1) in low, middle, and high tertiles of PTFQI index, respectively (p-value <0.001). In addition, the high TSHI and TT4RI tertile participants were younger and had lower WC and higher TSH levels.

**Table 1:** Baseline characteristics of the population based on PTFQI tertiles.

	Total	Low PTFQI (1^st^ tertile)(-1, -0.05)	Medium PTFQI (2^nd^ tertile)(-0.05,0.2)	High PTFQI (3^rd^ tertile)(0.2-0.99)	p-value
	N=4,356	N=1,452	N=1,452	N=1,452	
Categorical Variables					
**Sex**	1,874 (43.0%)	642 (44.2%)	529 (36.5%)	703 (48.4%)	<0.001
**Education level**					<0.001
Primary school	1,596 (36.6%)	602 (41.4%)	572 (39.4%)	422 (29.1%)	
High school	1,730 (39.7%)	544 (37.4%)	553 (38.1%)	633 (43.6%)	
Diploma	1,030 (23.6%)	307 (21.1%)	326 (22.5%)	397 (27.3%)	
**Current Smoker**	482 (11.1%)	166 (11.4%)	138 (9.5%)	178 (12.3%)	0.072
**Low physical activity**	2,890 (66.3%)	966 (66.5%)	995 (68.6%)	929 (64.0%)	0.029
**Family history of diabetes**	1,400 (32.1%)	513 (35.3%)	465 (32.0%)	422 (29.1%)	0.002
**Glycemic status**					<0.001
Normoglycemia	3,068 (70.4%)	943 (64.9%)	1,021 (70.4%)	1,104 (76.0%)	
Prediabetes	1,288 (29.6%)	510 (35.1%)	430 (29.6%)	348 (24.0%)	
Continuous Variables					
**Age (yrs)**	46.4 ±13.7	49.0 ±13.2	47.0 ±13.7	43.3 ±13.7	<0.001
**Free T4 (ng/dl)**	1.2 ±0.3	1.1 ±0.1	1.1 ±0.5	1.3 ±0.1	<0.001
**Body mass index (kg/m^2^)**	28.0 ±4.7	28.3 ±4.6	28.3 ±4.8	27.3 ±4.6	<0.001
**Waist circumference (cm)**	94.3 ±11.5	95.4 ±11.3	94.7 ±11.5	92.8 ±11.6	<0.001
**Systolic blood pressure (mmHg)**	115.9 ±17.4	117.0 ±18.1	116.5 ±17.5	114.3 ±16.4	<0.001
**Diastolic blood pressure(mmHg)**	77.0 ±10.8	77.4 ±11.1	76.8 ±10.7	76.8 ±10.7	0.24
**Fasting plasma glucose (mg/dL)**	94.3 ±8.8	95.1 ±8.9	94.2 ±8.8	93.4 ±8.6	<0.001
**2hPCG (mg/dL)**	105.2 ±28.1	107.8 ±28.6	106.3 ±28.2	101.4 ±27.0	<0.001
**Triglycerides (mg/dL)**	142.8 ±87.1	148.8 ±92.3	145.6 ±86.6	134.2 ±81.4	<0.001
**Total cholesterol (mg/dL)**	192.1 ±38.3	195.7 ±39.5	193.7 ±38.5	186.8 ±36.2	<0.001
**TSH (mU/L)**	2.1 (1.4-3.4)	1.4 (1.0-1.8)	2.7 (1.7-4.4)	2.9 (2.1-4.1)	<0.001
**Anti-TPO (IU/mL)**	5.8 (3.4-12.4)	4.9 (3.0-8.8)	6.5 (3.5-19.6)	6.2 (3.7-13.9)	<0.001

The categorical and continuous variables were reported as count (percentage), mean ± SD, and median (IQR), respectively. The first, second, and third tertiles of PTFQI were described as low, medium, and high PTFQI groups, respectively. Thyroid hormone sensitivity was calculated and represented using PTFQI (Parametric Thyroid Feedback Quantile-based Index).

**Figure 2:**
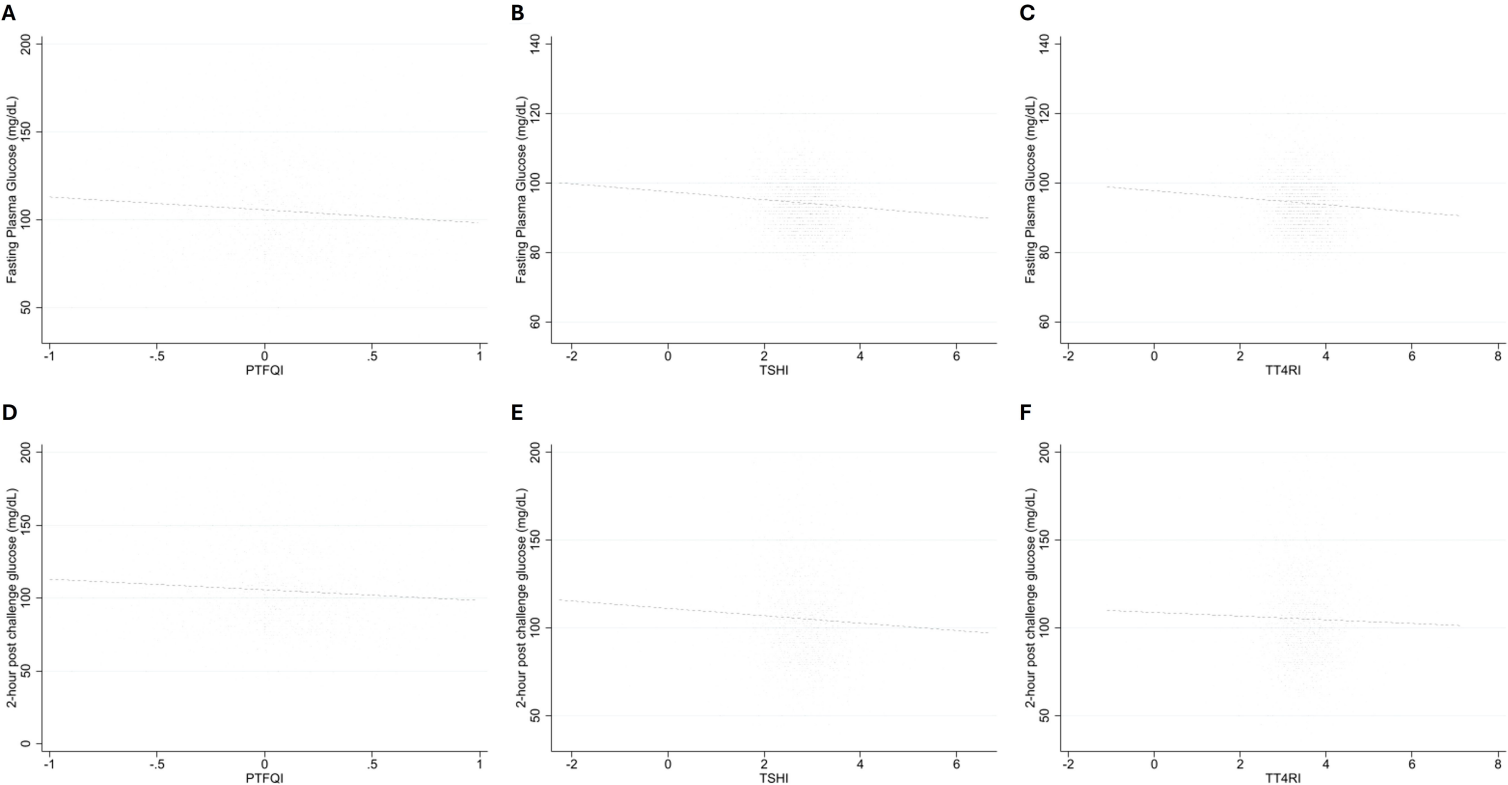
The correlation between fasting plasma glucose and **(A)** PTFQI, **(B)** TSHI, **(C)** lnTT4RI, and 2-hour post-challenge plasma glucose and **(D)** PTFQI, **(E)** TSHI, **(F)** lnTT4RI. Thyroid hormone sensitivity was calculated and represented using the following indices: PTFQI (Parametric Thyroid Feedback Quantile-based Index), TSHI (Thyroid-stimulating hormone index), TT4RI (Thyrotroph T4 Resistance Index). PTFQI, Parametric Thyroid Feedback Quantile-based Index; TSHI, Thyroid-stimulating hormone index; TT4RI, Thyrotroph T4 Resistance Index. To better illustrate the graphs, the participants with thyroid sensitivity indices three times below and above the standard deviation values of the study population were not displayed. Outliers were excluded from the figure for visual clarity but were included in all statistical analyses. Since the results did not change meaningfully when excluding these outliers, they were retained in the final analysis to preserve the full variability of the data.

Regarding glycemic status, 1288 (29.6%) individuals were prediabetic and 3068 (70.4%) had normal glycemic status. The prevalence of prediabetes was lower in higher tertiles of PTFQI, TT4RI, and TSHI indices (p-values<0.001). There was a negative correlation between FPG and all thyroid hormone sensitivity indices (PTFQI: r = -0.094, TSHI: -0.1, and lnTT4RI: r = -0.098) (**Figures 2A–C**). There was also a negative correlation between 2-hPCG and all thyroid hormone sensitivity indices (PTFQI: r = -0.096, TSHI: -0.054, and lnTT4RI: r = -0.031) (**Figures 2D–F**).

### Thyroid hormone sensitivity indices based on thyroid function

Participants were divided into hypothyroidism, euthyroidism, and hyperthyroidism groups based on their thyroid function. The mean/median of indices showed a significant reduction from hypothyroidism to euthyroidism and from euthyroidism to hyperthyroidism. The mean PTFQI index in each group is as follows: 0.2 ± 0.2 (hypothyroidism), 0.1 ± 0.3 (euthyroidism), and -0.3 ± 0.3 (hyperthyroidism) (*P <*0.001) (**Supplementary Table 3**). Moreover, **Supplementary Table 4** summarizes the mean/median of these indices in overt hypothyroidism, subclinical hypothyroidism, euthyroidism, subclinical hyperthyroidism, and overt hyperthyroidism groups.

### Association between thyroid hormone sensitivity indices and prediabetes

**Table 2** represents ORs and 95% CIs for prediabetes per 1-SD increase of PTFQI, TSHI, and lnTT4RI in the total population. One SD increase in PTFQI, TSHI, and lnTT4RI index was significantly associated with lower odds of prediabetes in all models. In the fully adjusted model, there was still a significant association between PTFQI, TSHI, and lnTT4RI and prediabetes (OR: 0.88 (95%CI: 0.82-0.94) for a 1-SD increase in PTFQI; OR: 0.84 (95%CI: 0.79-0.93) for 1-SD increase in TSHI; OR: 0.88 (95%CI: 0.81-0.94) for 1-SD increase in TT4RI). In the euthyroid subgroup of the total population, the ORs for prediabetes per one unit increase in PTFQI, TSHI, and lnTT4RI were 0.89 (95%CI: 0.83-0.97), 0.83 (95%CI: 0.74-0.94), and 0.83 (95%CI: 0.74-0.93) (**Table 3**).

**Table 2:** Odds ratio (95%CI) of prediabetes per 1 SD increase in the thyroid hormone sensitivity indices.

	Odds ratio (95%CI) of prediabetes				
	Crude	Model 1	Model 2	Model 3	Model 4
**PTFQI**	**0.78 (0.73-0.83)**	**0.85 (0.79-0.91)**	**0.84 (0.79-0.91)**	**0.87 (0.81-0.93)**	**0.88 (0.82-0.94)**
**TSHI**	**0.8 (0.74-0.86)**	**0.88 (0.81-0.95)**	**0.87 (0.8-0.94)**	**0.87 (0.8-0.94)**	**0.86 (0.79-0.93)**
**Ln TT4RI**	**0.84 (0.79-0.9)**	**0.91 (0.85-0.97)**	**0.9 (0.84-0.97)**	**0.89 (0.83-0.96)**	**0.88 (0.81-0.94)**

Model 1: Adjusted for age and sex., Model 2: Adjusted for age, sex, and anti-TPO., Model 3: Adjusted for age, sex, anti-TPO, education level, smoking status, family history of diabetes, BMI, and physical activity, Model 4: Adjusted for age, sex, anti-TPO, education level, smoking status, family history of diabetes, BMI, physical activity, HDL cholesterol, triglyceride, and total cholesterol.

Thyroid hormone sensitivity was calculated and represented using the following indices: PTFQI (Parametric Thyroid Feedback Quantile-based Index), TSHI (Thyroid-stimulating hormone index), TT4RI (Thyrotroph T4 Resistance Index).

**Table 3:** Odds ratio (95%CI) of prediabetes per 1 SD increase in the thyroid hormone sensitivity indices in the euthyroid population.

	Odds ratio (95%CI) of prediabetes				
	Crude	Model 1	Model 2	Model 3	Model 4
**PTFQI**	**0.78 (0.73-0.84)**	**0.86 (0.79-0.92)**	**0.85 (0.79-0.92)**	**0.88 (0.81-0.95)**	**0.89 (0.83-0.97)**
**TSHI**	**0.7 (0.63-0.78)**	**0.81 (0.73-0.91)**	**0.81 (0.72-0.91)**	**0.82 (0.73-0.92)**	**0.83 (0.74-0.94)**
**Ln TT4RI**	**0.73 (0.66-0.82)**	**0.84 (0.75-0.94)**	**0.83 (0.75-0.93)**	**0.83 (0.74-0.93)**	**0.83 (0.74-0.93)**

Model 1: Adjusted for age and sex; Model 2: Adjusted for age, sex, and anti-tpo; Model 3: Adjusted for age, sex, anti-tpo, education level, smoking status, family history of diabetes, BMI, and physical activity; Model 4: Adjusted for age, sex, anti-tpo, education level, smoking status, family history of diabetes, BMI, physical activity, HDL cholesterol, triglyceride, and total cholesterol.

Thyroid hormone sensitivity was calculated and represented using the following indices: PTFQI (Parametric Thyroid Feedback Quantile-based Index), TSHI (Thyroid-stimulating hormone index), TT4RI (Thyrotroph T4 Resistance Index).

We also evaluated the association of thyroid hormone sensitivity indices using the tertile approach. The odds ratios for the risk of prediabetes in the general population based on tertiles of PTFQI, TSHI, and TT4RI indices are also available in **Supplementary Tables 5**, **6**. Using the PTFQI index, the odds of prediabetes were significantly lower in the medium (OR= 0.82; 95%CI: 0.69–0.98) and high PTFQI (OR= 0.74; 95%CI: 0.62–0.89) group compared to the low PTFQI group. The participants with high TSHI and TT4RI also had a significantly decreased risk of prediabetes compared to their reference groups. The associations were consistent in the euthyroid subjects for tertiles of PTFQI, TSHI, and TT4RI indices (**Supplementary Table 6**).

### Subgroup analysis

The association between PFTQI, TSHI, and TT4RI indices and the odds of prediabetes in different subgroups was evaluated (**Table 4**). The association was significant in age subgroups and both obese and non-obese participants. However, in other subgroups, the association was only observed in women, anti-TPO-negative subjects, and non-smokers. The odds of prediabetes in tertiles of PFTQI, TSHI, and TT4RI in different subgroups are also presented in **Supplementary Tables 7–9**.

**Table 4:** Odds ratio (95%CI) of prediabetes per 1 SD increase in the thyroid hormone sensitivity indices in different subgroups.

	Odds ratio (95%CI) of prediabetes		
	TSHI	Ln TT4RI	PTFQI
Sex			
Male	0.89 (0.78-1)	0.91 (0.81-1.02)	0.91 (0.83-1.01)
Female	**0.83 (0.74-0.93)**	**0.85 (0.77-0.93)**	**0.83 (0.75-0.93)**
Age			
<45 years	**0.83 (0.72-0.95)**	**0.86 (0.76-0.98)**	**0.84 (0.75-0.94)**
≥45 years	**0.84 (0.76-0.93)**	**0.85 (0.78-0.93)**	**0.89 (0.81-0.97)**
Anti-TPO			
Negative	**0.82 (0.75-0.9)**	**0.83 (0.76-0.91)**	**0.87 (0.8-0.93)**
Positive	0.96 (0.84-1.1)	1 (0.87-1.16)	0.99 (0.78-1.25)
Smoking			
No	**0.84 (0.77-0.91)**	**0.85 (0.79-0.92)**	**0.87 (0.81-0.94)**
Yes	0.98 (0.84-1.16)	1.05 (0.85-1.3)	0.91 (0.74-1.13)
Obesity			
Non-obese	**0.86 (0.78-0.95)**	**0.88 (0.8-0.96)**	**0.88 (0.8-0.96)**
Obese	**0.84 (0.73-0.97)**	**0.86 (0.75-0.98)**	**0.86 (0.76-0.97)**

Adjusted for age, sex, anti-TPO, education level, smoking status, family history of diabetes, BMI, physical activity, HDL cholesterol, triglyceride, and total cholesterol.

Thyroid hormone sensitivity was calculated and represented using the following indices: PTFQI (Parametric Thyroid Feedback Quantile-based Index), TSHI (Thyroid-stimulating hormone index), TT4RI (Thyrotroph T4 Resistance Index).

## Discussion

The current population-based study is among the first studies investigating the association between prediabetes and thyroid hormone sensitivity in a relatively large sample size. There was a negative correlation between FPG and thyroid hormone resistance. Also, decreased central thyroid hormone sensitivity, as presented by increased TSHI, TT4RI, and PTFQI, was associated with a lower risk of prediabetes even in euthyroid individuals, those without thyroid autoimmunity, and in normal, overweight, and obese individuals. The association was not observed in men, smokers, and TPOAb-positive individuals. Thyroid hormones play a crucial role in regulating insulin resistance and glucose homeostasis. Many studies have demonstrated a significant association between thyroid dysfunction and thyroid hormones and type 2 diabetes mellitus (T2DM) or prediabetes (19, 20). More consistently, hypothyroidism was associated with diabetes and FBS (20–24). However, the association of higher free thyroid hormones (FT3/FT4) with the incidence of diabetes has been reported (25–27). In the population-based Tehran thyroid Study, the longitudinal trend of serum thyroid hormones toward hyperthyroidism, rising TSH, and decreasing ft4 serum values over 10 years were associated with the development of T2DM, using a joint modeling approach (28, 29). The association of higher serum TSH and free thyroid hormones with diabetes is inconsistent with the mechanism of regulating thyroid hormone through a negative feedback loop in the hypothalamus-pituitary–thyroid (HPT). These findings suggest that any deviation from the normal range of thyroid hormone levels, whether hypo or hyperthyroid, may contribute to an increased risk of glycemic dysregulation. Previous research inconsistencies emphasize that TSH or thyroid hormone levels alone may not fully account for the connection between the thyroid system and glycemic dysregulation. Based on the conflicting data regarding the supposed negative thyroid feedback loop and mixed findings on the metabolic impacts of hypo and hyperthyroidism, it is suggested that the simultaneous presence of high TSH and high thyroid hormones could indicate a form of mild acquired resistance to thyroid hormones. This could help explain the unexpected and controversial findings.

While several studies have assessed the association between thyroid hormone sensitivity and diabetes (9, 17, 30), its association with prediabetes is scarcely investigated in the literature (31). Our findings align with emerging evidence on thyroid-glucose interplay while highlighting unique aspects. The inverse association between central thyroid resistance indices (PTFQI, TSHI, TT4RI) and prediabetes corroborates Liu et al.’s (31) cross-sectional study in China (n=4,378), where higher PTFQI (reflecting reduced sensitivity) was linked to lower prediabetes risk (OR: 0.85, 95% CI: 0.76–0.95). This relationship is further supported by two key studies: (1) Yu et al.’s (32) multicenter retrospective analysis of 30,244 coronary heart disease patients, which demonstrated negative associations between central resistance indices (TSHI, TT4RI, PTFQI) and elevated blood glucose (EBG) risk (p<0.01 for trend), and (2) Liu et al.’s (33) longitudinal study of 2,927 pregnant women in South China, where increased central/peripheral thyroid resistance during early pregnancy predicted reduced gestational diabetes risk (OR: 0.72, 95% CI: 0.56–0.93). These findings collectively suggest a protective role of thyroid hormone resistance in early dysglycemia stages. However, our results contrast with Laclaustra et al. (9), who reported impaired sensitivity (higher indices) predicting diabetes in euthyroid individuals (HR: 1.35, 95% CI: 1.12–1.63). This discrepancy may reflect differences in: (i) outcome definitions (prediabetes *vs*. diabetes), as compensatory FT4 elevation in early metabolic dysfunction may transiently improve insulin sensitivity via TRβ-mediated mitochondrial uncoupling (33); and (ii) population characteristics (e.g., our cohort’s younger age [46.4 ± 13.7 years] *vs*. Laclaustra’s [55.7 ± 8.7 years]). Mechanistically, animal studies confirm TRβ activation enhances skeletal muscle glucose uptake (34), aligning with our observed negative correlations between resistance indices and FPG (r=−0.094 to −0.1).

The inverse association between thyroid resistance and prediabetes aligns with evidence that reduced thyroid hormone action may lower hepatic gluconeogenesis and improve peripheral insulin sensitivity (35). Other pathways include the insulin signaling pathway, genes regulating insulin resistance, and beta-cell proliferation (36), and hormonal regulation (leptin) interaction between the HPT axis and glucose metabolism (35). Most previous studies showed the positive association of serum TSH and the negative association of the level of FT3 and FT4 with insulin resistance. High serum TSH values in clinical and subclinical hypothyroidism levels were associated with comparable insulin resistance due to impaired translocation of GLUT4 glucose transporters on the plasma membrane, leading to decreased insulin-stimulated glucose disposal in muscle and adipose tissue. Individuals with lower central sensitivity to thyroid hormones often exhibit higher serum FT4 levels, potentially reducing the likelihood of developing prediabetes by enhancing insulin sensitivity and glucose utilization. Leptin is secreted by adipose tissue and regulates caloric intake, and energy storage may interact between the HPT axis and glucose metabolism. Changes in leptin levels have been observed in thyroid dysfunction, affecting feeding behavior, adiposity, and glucose metabolism (35, 37). The mechanisms linking central thyroid hormone sensitivity and the leptin pathway remain unclear.

While our cross-sectional study design precludes causality, alterations in glucose metabolism may influence thyroid hormone sensitivity, potentially disrupting thyroid physiology and, in turn, affecting glucose homeostasis. A 10-year cohort study in China on 7283 participants found no evidence that thyroid hormone sensitivity status could predict diabetes development in euthyroid people, and an increased fasting glucose level preceded reduced sensitivity to thyroid hormones (38). The reciprocal relationship between glucose metabolism and thyroid function implies that altered thyroid function may also impact glucose metabolism, warranting further investigation using longitudinal designs or experimental manipulations to clarify the mechanisms underlying this intricate association.

Sex differences in thyroid hormone sensitivity and metabolism may explain the stronger association between thyroid resistance indices and prediabetes observed in women. Women have higher levels of thyroid-binding globulin due to estrogen, which affects total thyroid hormone measurements but not necessarily free hormone levels or tissue sensitivity (39). Moreover, Estrogen has been shown to enhance central sensitivity to thyroid hormones via effects on hypothalamic-pituitary-thyroid (HPT) axis regulation (40). This could mean that subtle changes in thyroid hormone resistance might manifest differently in men, who lack this estrogen-mediated modulation, potentially explaining why no significant associations were observed in male participants. Also, there are notable differences in adipose tissue distribution and insulin sensitivity between the sexes with women tending to have greater subcutaneous fat deposition, while men accumulate more visceral adiposity—a known risk factor for insulin resistance and prediabetes. Visceral fat is more metabolically active and has been shown to promote inflammation and hepatic insulin resistance, which may modify the relationship between thyroid hormone signaling and glucose homeostasis (41).

Our study demonstrates an inverse association between central thyroid hormone sensitivity indices and prediabetes, particularly in euthyroid and anti-TPO-negative individuals. The absence of this association in anti-TPO-positive subjects suggests that thyroid autoimmunity may disrupt the metabolic effect of reduced thyroid hormone sensitivity, possibly through mechanisms involving low-grade inflammation or subtle thyroid dysfunction (42, 43). Anti-TPO antibodies, markers of autoimmune thyroiditis, have been linked to altered glucose metabolism in some studies, though results remain inconsistent (44). This highlights the need to consider autoimmune status when evaluating thyroid-glucose interactions, as anti-TPO positivity may identify a distinct subgroup with divergent metabolic responses.

This study is strengthened by its population-based design on a large sample size, which evaluated a novel idea assessing thyroid hormone sensitivity with prediabetes. Moreover, we investigated these relations in euthyroid patients as well as several subgroups of them that could provide researchers with a better understanding of the possible association and pathophysiology. While our findings suggest that decreased central thyroid sensitivity is associated with lower prediabetes risk, causality cannot be inferred due to the cross-sectional design. Unmeasured confounders (e.g., cortisol, glucagon, insulin) or residual thyroid-independent effects may contribute, though our adjustments for metabolic proxies (which correlate with insulin resistance and hormonal dysregulation) e.g. obesity, lipids, and thyroid autoimmunity, mitigate some concerns. Prospective studies with repeated thyroid/glucose measurements and mediation analyses are needed to confirm whether thyroid resistance directly mitigates diabetes risk or serves as a marker of other protective metabolic adaptations. We lacked data on the menstrual cycle phase and sex hormone levels (estradiol, progesterone) that are known to modulate both thyroid function and glucose homeostasis (45). While our large sample size may partially average out these effects, residual confounding remains possible. The exclusion of individuals on thyroid/antidiabetic medications was necessary to minimize confounding and isolate the intrinsic relationship between thyroid hormone sensitivity indices and prediabetes. While this enhances internal validity, it may limit generalizability to treated or clinically complex populations, as medication effects (e.g., levothyroxine or metformin altering thyroid/glucose metabolism) and disease-specific hormonal interactions were not captured. However, our findings remain highly applicable to the general euthyroid population, aligning with the study’s primary aim. Future studies should validate these associations in cohorts with medication use or overt thyroid disease, where altered thyroid-glucose interactions may differ. In this study, fT3 was not routinely measured due to cost constraints and lower clinical utility for population screening compared to TSH/fT4. Also, Peripheral conversion (via deiodinases) is influenced by non-thyroidal factors (e.g., inflammation, malnutrition) that could confound sensitivity indices. Therefore, it seems that our focus on central resistance (pituitary-thyroid feedback) is adequately captured by TSH-fT4 dynamics.

The results of the current study could give better insight into the inevitable role of metabolic dysfunction in thyroid hormone sensitivity. Our findings suggest that decreased central sensitivity to thyroid hormones could be a protective factor for prediabetes even in euthyroid and TPOAb-negative individuals. Our study offers a novel framework by introducing thyroid hormone sensitivity indices (PTFQI, TSHI, and TT4RI) as clinically useful tools to evaluate the role of central thyroid resistance in glucose metabolism, even in euthyroid individuals. These indices, calculated from routine thyroid function tests, may identify subpopulations at altered metabolic risk, bridging a gap between traditional thyroid diagnostics and glucose homeostasis. While promising, their prognostic utility warrants validation in longitudinal and interventional studies.

## Future directions

Prospective studies should investigate whether changes in thyroid sensitivity indices precede prediabetes onset, leveraging longitudinal designs to establish causality. Incorporating FT3 measurements could refine peripheral sensitivity assessments, while mechanistic research should explore tissue-specific thyroid actions (e.g., via GLUT4 or leptin pathways (35). Stratification by anti-TPO status and integration of multi-omics data (e.g., metabolomics) may uncover shared pathways, offering insights for targeted prevention strategies.

## Data Availability

The raw data supporting the conclusions of this article will be made available by the authors, without undue reservation.
