# Supplementary material for: Association between central thyroid hormone sensitivity and prediabetes: Tehran thyroid study
Source: Front Endocrinol (Lausanne). 2025 Jun 17;16:1534058. doi: 10.3389/fendo.2025.1534058 (PMC12208861; doi:10.3389/fendo.2025.1534058)
Supplement: Supplementary file 1 [file Supplementaryfile1.docx]

**Supplementary Table 1.** Baseline characteristics of the population based on TSHI tertiles

|  | **Total** | **Low TSHI**  **(-3.38,2.55)** | **Medium TSHI**  **(2.55-3.11)** | **High TSHI**  **(3.11-29.27)** | **p-value** |
| --- | --- | --- | --- | --- | --- |
|  | N=4,356 | N=1,452 | N=1,452 | N=1,452 |  |
| **Age (yrs)** | 46.4±13.7 | 49.0±13.5 | 45.8±13.7 | 44.5±13.6 | <0.001 |
| **Male** | 1,874 (43.0%) | 697 (48.0%) | 647 (44.6%) | 530 (36.5%) | <0.001 |
| **Education level** |  |  |  |  | <0.001 |
| **Primary school** | 1,596 (36.6%) | 602 (41.5%) | 504 (34.7%) | 490 (33.7%) |  |
| **High school** | 1,730 (39.7%) | 534 (36.8%) | 576 (39.7%) | 620 (42.7%) |  |
| **Diploma** | 1,030 (23.6%) | 316 (21.8%) | 372 (25.6%) | 342 (23.6%) |  |
| **Current Smoker** | 482 (11.1%) | 193 (13.3%) | 155 (10.7%) | 134 (9.2%) | 0.012 |
| **Low physical activity** | 2,890 (66.3%) | 954 (65.7%) | 949 (65.4%) | 987 (68.0%) | 0.093 |
| **Family history of diabetes** | 1,400 (32.1%) | 497 (34.2%) | 476 (32.8%) | 427 (29.4%) | 0.017 |
| **TSH (mU/L)** | 2.1 (1.4-3.4) | 1.2 (0.9-1.6) | 2.2 (1.8-2.7) | 4.1 (3.1-5.8) | <0.001 |
| **Free T4 (ng/dl)** | 1.2±0.3 | 1.1±0.2 | 1.2±0.2 | 1.2±0.5 | <0.001 |
| **Anti-TPO (IU/mL)** | 5.8 (3.4-12.4) | 4.9 (3.0-8.9) | 5.3 (3.3-9.8) | 8.1 (4.1-41.9) | <0.001 |
| **Body mass index (kg/m^2^)** | 28.0±4.7 | 28.2±4.5 | 27.8±4.6 | 27.9±4.9 | 0.13 |
| **Waist circumference (cm)** | 94.3±11.5 | 95.5±11.2 | 93.8±11.4 | 93.6±11.9 | <0.001 |
| **Systolic blood pressure (mmHg)** | 115.9±17.4 | 117.2±18.0 | 116.0±17.6 | 114.6±16.4 | <0.001 |
| **Diastolic blood pressure(mmHg)** | 77.0±10.8 | 77.4±11.1 | 77.1±10.6 | 76.5±10.8 | 0.092 |
| **Fasting plasma glucose (mg/dL)** | 94.3±8.8 | 95.4±8.8 | 94.0±8.9 | 93.4±8.6 | <0.001 |
| **2hPCG (mg/dL)** | 105.2±28.1 | 107.2±28.9 | 105.0±28.3 | 103.3±26.9 | <0.001 |
| **Triglycerides (mg/dL)** | 142.8±87.1 | 147.6±92.4 | 141.9±84.0 | 138.9±84.5 | 0.024 |
| **Total cholesterol (mg/dL)** | 192.1±38.3 | 194.7±39.5 | 190.7±37.5 | 190.9±37.7 | 0.007 |
| **Glycemic status** |  |  |  |  | <0.001 |
| **Normoglycemia** | 3,068 (70.4%) | 940 (64.7%) | 1,037 (71.4%) | 1,091 (75.1%) |  |
| **Prediabetes** | 1,288 (29.6%) | 512 (35.3%) | 415 (28.6%) | 361 (24.9%) |  |

The categorical and continuous variables were reported as count (percentage) and mean ± SD, respectively.

The first, second, and third tertiles of TSHI were described as low, medium, and high TSHI groups, respectively.

Abbreviations: TSHI, thyroid-stimulating hormone index; TSH, thyroid stimulating hormone; Anti-TPO, anti-thyroid peroxidase; 2-hPCG**,** 2 h post-challenge plasma glucose

**Supplementary Table 2.** Baseline characteristics of the population based on TT4RI tertiles

|  | **Total** | **Low Ln(TT4RI)**  **(-2.64,3.23)** | **Medium Ln(TT4RI)**  **(3.23,3.65)** | **High Ln(TT4RI)**  **(3.76,7.15)** | **p-value** |
| --- | --- | --- | --- | --- | --- |
|  | N=4,356 | N=1,452 | N=1,452 | N=1,452 |  |
| **Age (yrs)** | 46.4±13.7 | 48.7±13.7 | 45.8±13.5 | 44.8±13.6 | <0.001 |
| **Male** | 1,874 (43.0%) | 739 (50.9%) | 649 (44.7%) | 486 (33.5%) | <0.001 |
| **Education level** |  |  |  |  | 0.001 |
| **Primary school** | 1,596 (36.6%) | 589 (40.6%) | 495 (34.1%) | 512 (35.3%) |  |
| **High school** | 1,730 (39.7%) | 528 (36.4%) | 593 (40.8%) | 609 (41.9%) |  |
| **Diploma** | 1,030 (23.6%) | 335 (23.1%) | 364 (25.1%) | 331 (22.8%) |  |
| **Current Smoker** | 482 (11.1%) | 206 (14.2%) | 162 (11.2%) | 114 (7.9%) | <0.001 |
| **Low physical activity** | 2,890 (66.3%) | 950 (65.4%) | 949 (65.4%) | 991 (68.3%) | 0.053 |
| **Family history of diabetes** | 1,400 (32.1%) | 479 (33.0%) | 484 (33.3%) | 437 (30.1%) | 0.12 |
| **TSH (mU/L)** | 2.1 (1.4-3.4) | 1.2 (0.9-1.4) | 2.1 (1.9-2.5) | 4.2 (3.3-5.8) | <0.001 |
| **Free T4 (ng/dl)** | 1.2±0.3 | 1.2±0.5 | 1.2±0.2 | 1.2±0.2 | <0.001 |
| **Anti-TPO (IU/mL)** | 5.8 (3.4-12.4) | 4.8 (3.0-8.6) | 5.3 (3.3-10.0) | 8.4 (4.1-48.3) | <0.001 |
| **Body mass index (kg/m^2^)** | 28.0±4.7 | 28.0±4.5 | 27.9±4.6 | 28.1±4.9 | 0.49 |
| **Waist circumference (cm)** | 94.3±11.5 | 95.1±11.2 | 94.0±11.3 | 93.7±11.9 | 0.002 |
| **Systolic blood pressure (mmHg)** | 115.9±17.4 | 117.2±18.2 | 116.1±17.6 | 114.6±16.3 | <0.001 |
| **Diastolic blood pressure(mmHg)** | 77.0±10.8 | 77.3±11.0 | 77.2±10.7 | 76.5±10.8 | 0.073 |
| **Fasting plasma glucose (mg/dL)** | 94.3±8.8 | 95.3±8.7 | 94.1±9.0 | 93.4±8.7 | <0.001 |
| **2-hPCG (mg/dL)** | 105.2±28.1 | 106.5±29.0 | 105.2±28.2 | 103.8±27.0 | 0.034 |
| **Triglycerides (mg/dL)** | 142.8±87.1 | 145.6±90.9 | 143.7±87.4 | 139.2±82.7 | 0.13 |
| **Total cholesterol (mg/dL)** | 192.1±38.3 | 193.9±39.5 | 190.5±37.8 | 191.8±37.5 | 0.060 |
| **Glycemic status** |  |  |  |  | <0.001 |
| **Normoglycemia** | 3,068 (70.4%) | 951 (65.5%) | 1,033 (71.1%) | 1,084 (74.7%) |  |
| **Prediabetes** | 1,288 (29.6%) | 501 (34.5%) | 419 (28.9%) | 368 (25.3%) |  |

The categorical and continuous variables were reported as count (percentage) and mean ± SD, respectively.

The first, second, and third tertiles of TT4RI were described as low, medium, and high TT4RI groups, respectively.

Abbreviations: TT4RI, Thyrotroph T4 Resistance Index; TSH, thyroid stimulating hormone; Anti-TPO, anti-thyroid peroxidase; 2-hPCG**,** 2 h post-challenge plasma glucose

**Supplementary Table 3.** Mean or median of thyroid hormone sensitivity indices in each thyroid function group

|  | **Total** | **Hypothyroidism** | **Euthyroidism** | **Hyperthyroidism** | **p-value** |
| --- | --- | --- | --- | --- | --- |
|  | N=4,356 | N=478 | N=3,821 | N=57 |  |
| **PTFQI** | 0.1±0.3 | 0.2±0.2 | 0.1±0.3 | -0.3±0.3 | <0.001 |
| **TSHI** | 2.8±0.9 | 3.9±0.5 | 2.7±0.6 | 0.3±4.1 | <0.001 |
| **TT4RI** | 32.7 (21.7-50.5) | 98.8 (82.8-132.4) | 30.4 (21.0-43.7) | 1.8 (0.3-3.8) | <0.001 |

Data is represented as mean±SD or median (IQR).

Abbreviations: PTFQI, Parametric Thyroid Feedback Quantile-based Index; TSHI, Thyroid-stimulating hormone index; TT4RI, Thyrotroph T4 Resistance Index.

**Supplementary Table 4.** Mean or median of thyroid hormone sensitivity indices in each thyroid function group (overt and subclinical included)

|  | **Total** | **Overt hypothyroidism** | **Subclinical hypothyroidism** | **Euthyroidism** | **Subclinical hyperthyroidism** | **Over hyperthyroidism** | **p-value** |
| --- | --- | --- | --- | --- | --- | --- | --- |
|  | N=4,356 | N=110 | N=368 | N=3,821 | N=38 | N=19 |  |
| **PTFQI** | 0.1±0.3 | 0.0±0.0 | 0.3±0.2 | 0.1±0.3 | -0.5±0.3 | -0.0±0.0 | <0.001 |
| **TSHI** | 2.8±0.9 | 4.0±0.8 | 3.9±0.4 | 2.7±0.6 | -0.1±1.1 | 1.0±7.0 | <0.001 |
| **TT4RI** | 32.7 (21.7-50.5) | 133.1 (80.5-197.3) | 95.7 (83.0-117.8) | 30.4 (21.0-43.7) | 2.6 (0.8-3.8) | 0.3 (0.1-1.5) | <0.001 |

Data is represented as mean±SD or median (IQR).

Abbreviations: PTFQI, Parametric Thyroid Feedback Quantile-based Index; TSHI, Thyroid-stimulating hormone index; TT4RI, Thyrotroph T4 Resistance Index.

**Supplementary Table 5. Odds ratio (95%CI) of prediabetes according to the tertiles of thyroid hormone sensitivity indices**

| Prediabetes | Crude | Model 1 | Model 2 | Model 3 | Model 4 |
| --- | --- | --- | --- | --- | --- |
| PTFQI Tertiles | | | | | |
| Low PTFQI | Reference (1.00) | Reference (1.00) | Reference (1.00) | Reference (1.00) | Reference (1.00) |
| Medium PTFQI | **0.78 (0.67-0.91)** | **0.85 (0.72-1)** | **0.83 (0.71-0.98)** | **0.84 (0.71-0.99)** | **0.82 (0.69-0.98)** |
| High PTFQI | **0.58 (0.5-0.69)** | **0.7 (0.59-0.83)** | **0.7 (0.59-0.83)** | **0.73 (0.61-0.86)** | **0.74 (0.62-0.89)** |
| TSHI Tertiles | | | | | |
| Low TSHI | Reference (1.00) | Reference (1.00) | Reference (1.00) | Reference (1.00) | Reference (1.00) |
| Medium TSHI | **0.73 (0.63-0.86)** | **0.83 (0.7-0.98)** | **0.83 (0.7-0.97)** | **0.84 (0.71-0.99)** | **0.83 (0.7-0.98)** |
| High TSHI | **0.61 (0.52-0.71)** | **0.73 (0.62-0.87)** | **0.71 (0.6-0.84)** | **0.72 (0.61-0.86)** | **0.71 (0.59-0.85)** |
| TT4RI Tertiles | | | | | |
| Low TT4RI | Reference (1.00) | Reference (1.00) | Reference (1.00) | Reference (1.00) | Reference (1.00) |
| Medium TT4RI | **0.77 (0.66-0.9)** | 0.87 (0.74-1.02) | 0.87 (0.74-1.02) | 0.87 (0.73-1.02) | 0.85 (0.72-1.01) |
| High TT4RI | **0.64 (0.55-0.76)** | **0.77 (0.65-0.91)** | **0.75 (0.63-0.89)** | **0.75 (0.63-0.89)** | **0.73 (0.61-0.88)** |

Model 1: Adjusted for age and sex.
Model 2: Adjusted for age, sex, and anti-TPO.
Model 3: Adjusted for age, sex, anti-TPO, education level, smoking status, family history of diabetes, obesity, and physical activity.
Model 4: Adjusted for age, sex, anti-TPO, education level, smoking status, family history of diabetes, obesity (non-obese vs. obese), physical activity, HDL cholesterol (low vs. normal), triglyceride (normal vs. high), and total cholesterol (normal vs. high).

The first, second, and third tertiles of each index were described as low, medium, and high, respectively.

Abbreviations: PTFQI, Parametric Thyroid Feedback Quantile-based Index; TSHI, Thyroid-stimulating hormone index; TT4RI, Thyrotroph T4 Resistance Index.

**Supplementary Table 6. Odds ratio (95%CI) of prediabetes according to the tertiles of thyroid hormone sensitivity indices in the euthyroid population**

| Prediabetes | Crude | Model 1 | Model 2 | Model 3 | Model 4 |
| --- | --- | --- | --- | --- | --- |
| PTFQI Tertiles | | | | | |
| Low PTFQI | Reference (1.00) | Reference (1.00) | Reference (1.00) | Reference (1.00) | Reference (1.00) |
| Medium PTFQI | **0.78 (0.66-0.93)** | 0.86 (0.72-1.02) | 0.86 (0.72-1.02) | 0.87 (0.73-1.04) | 0.87 (0.72-1.04) |
| High PTFQI | **0.59 (0.5-0.7)** | **0.72 (0.6-0.86)** | **0.71 (0.6-0.86)** | **0.74 (0.62-0.89)** | **0.77 (0.64-0.93)** |
| TSHI Tertiles | | | | | |
| Low TSHI | Reference (1.00) | Reference (1.00) | Reference (1.00) | Reference (1.00) | Reference (1.00) |
| Medium TSHI | **0.77 (0.65-0.91)** | 0.84 (0.71-1) | 0.84 (0.71-1) | 0.86 (0.72-1.02) | 0.84 (0.7-1) |
| High TSHI | **0.58 (0.49-0.69)** | **0.71 (0.6-0.86)** | **0.71 (0.59-0.85)** | **0.73 (0.61-0.88)** | **0.73 (0.61-0.88)** |
| TT4RI Tertiles | | | | | |
| Low TT4RI | Reference (1.00) | Reference (1.00) | Reference (1.00) | Reference (1.00) | Reference (1.00) |
| Medium TT4RI | **0.83 (0.7-0.98)** | 0.93 (0.78-1.1) | 0.92 (0.78-1.1) | 0.92 (0.77-1.1) | 0.92 (0.77-1.1) |
| High TT4RI | **0.59 (0.49-0.7)** | **0.71 (0.59-0.85)** | **0.7 (0.59-0.85)** | **0.71 (0.59-0.85)** | **0.71 (0.58-0.85)** |

Model 1: Adjusted for age and sex.
Model 2: Adjusted for age, sex, and anti-TPO.
Model 3: Adjusted for age, sex, anti-TPO, education level, smoking status, family history of diabetes, obesity, and physical activity.
Model 4: Adjusted for age, sex, anti-TPO, education level, smoking status, family history of diabetes, obesity (non-obese vs. obese), physical activity, HDL cholesterol (low vs. normal), triglyceride (normal vs. high), and total cholesterol (normal vs. high).

The first, second, and third tertiles of each index were described as low, medium, and high, respectively.

Abbreviations: PTFQI, Parametric Thyroid Feedback Quantile-based Index; TSHI, Thyroid-stimulating hormone index; TT4RI, Thyrotroph T4 Resistance Index.

**Supplementary Table 7.** Subgroup analysis for risk of prediabetes based on PTFQI tertiles

|  | Low PTFQI | Medium PTFQI | High PTFQI |
| --- | --- | --- | --- |
| Sex |  |  |  |
| Male | Reference (1.00) | 1.11 (0.87-1.43) | 0.84 (0.66-1.07) |
| Female | Reference (1.00) | **0.63 (0.5-0.8)** | **0.66 (0.51-0.84)** |
| Age |  |  |  |
| <45 | Reference (1.00) | **0.7 (0.53-0.93)** | **0.71 (0.55-0.93)** |
| ≥45 | Reference (1.00) | 0.89 (0.72-1.1) | **0.73 (0.58-0.92)** |
| Anti-TPO |  |  |  |
| Negative | Reference (1.00) | 0.84 (0.7-1.01) | **0.72 (0.6-0.86)** |
| Positive | Reference (1.00) | 0.81 (0.47-1.41) | 0.95 (0.53-1.69) |
| Smoking |  |  |  |
| No | Reference (1.00) | **0.82 (0.68-0.98)** | **0.72 (0.6-0.87)** |
| Yes | Reference (1.00) | 1.03 (0.6-1.78) | 0.82 (0.49-1.38) |
| Obesity |  |  |  |
| Non-obese | Reference (1.00) | **0.78 (0.63-0.97)** | **0.75 (0.6-0.93)** |
| Obese | Reference (1.00) | 0.91 (0.69-1.2) | 0.75 (0.55-1.01) |

The first, second, and third tertiles of PTFQI were described as low, medium, and high PTFQI groups, respectively.

Abbreviations: Anti-TPO, anti-thyroid peroxidase; PTFQI, Parametric Thyroid Feedback Quantile-based Index.

**Supplementary Table 8.** Subgroup analysis for risk of prediabetes based on TSHI tertiles

|  | Low TSHI | Medium TSHI | High TSHI |
| --- | --- | --- | --- |
| Sex |  |  |  |
| Male | Reference (1.00) | 1.06 (0.84-1.35) | 0.82 (0.63-1.07) |
| Female | Reference (1.00) | **0.63 (0.5-0.81)** | **0.61 (0.48-0.78)** |
| Age |  |  |  |
| <45 | Reference (1.00) | **0.74 (0.57-0.97)** | **0.64 (0.49-0.84)** |
| ≥45 | Reference (1.00) | 0.88 (0.71-1.09) | **0.73 (0.58-0.92)** |
| Anti-TPO |  |  |  |
| Negative | Reference (1.00) | **0.81 (0.68-0.97)** | **0.68 (0.56-0.82)** |
| Positive | Reference (1.00) | 1.18 (0.63-2.21) | 1.05 (0.61-1.81) |
| Smoking |  |  |  |
| No | Reference (1.00) | **0.79 (0.66-0.94)** | **0.69 (0.57-0.84)** |
| Yes | Reference (1.00) | 1.28 (0.76-2.14) | 0.78 (0.45-1.38) |
| Obesity |  |  |  |
| Non-obese | Reference (1.00) | **0.79 (0.64-0.97)** | **0.7 (0.56-0.88)** |
| Obese | Reference (1.00) | 0.91 (0.68-1.21) | **0.74 (0.55-0.99)** |

The first, second, and third tertiles of TSHI were described as low, medium, and high TSHI groups, respectively.

Abbreviations: Anti-TPO, anti-thyroid peroxidase; TSHI, Thyroid-stimulating hormone index.

**Supplementary Table 9.** Subgroup analysis for risk of prediabetes based on TT4RI tertiles

|  | Low TT4RI | Medium TT4RI | High TT4RI |
| --- | --- | --- | --- |
| Sex |  |  |  |
| Male | Reference (1.00) | 1.07 (0.85-1.35) | 0.83 (0.64-1.09) |
| Female | Reference (1.00) | **0.66 (0.52-0.85)** | **0.63 (0.5-0.81)** |
| Age |  |  |  |
| <45 | Reference (1.00) | 0.8 (0.61-1.04) | **0.67 (0.51-0.89)** |
| ≥45 | Reference (1.00) | 0.87 (0.7-1.08) | **0.74 (0.58-0.93)** |
| Anti-TPO |  |  |  |
| Negative | Reference (1.00) | **0.82 (0.69-0.98)** | **0.7 (0.58-0.85)** |
| Positive | Reference (1.00) | 1.42 (0.74-2.73) | 1.18 (0.67-2.1) |
| Smoking |  |  |  |
| No | Reference (1.00) | **0.82 (0.69-0.98)** | **0.71 (0.58-0.86)** |
| Yes | Reference (1.00) | 1.04 (0.63-1.72) | 0.92 (0.52-1.65) |
| Obesity |  |  |  |
| Non-obese | Reference (1.00) | 0.85 (0.69-1.05) | **0.73 (0.58-0.92)** |
| Obese | Reference (1.00) | 0.84 (0.63-1.12) | **0.73 (0.54-0.98)** |

The first, second, and third tertiles of TT4RI were described as low, medium, and high TT4RI groups, respectively.

Abbreviations: Anti-TPO, anti-thyroid peroxidase;TT4RI, Thyrotroph T4 Resistance Index**.**
